# Physicochemical and Antioxidant Properties of Algerian Honey

**DOI:** 10.3390/molecules170911199

**Published:** 2012-09-20

**Authors:** Md. Ibrahim Khalil, Mohammed Moniruzzaman, Laïd Boukraâ, Mokhtar Benhanifia, Md. Asiful Islam, Md. Nazmul Islam, Siti Amrah Sulaiman, Siew Hua Gan

**Affiliations:** 1 Department of Pharmacology, School of Medical Sciences, Universiti Sains Malaysia, Kubang Kerian 16150, Kelantan, Malaysia; 2 Department of Biochemistry and Molecular Biology, Jahangirnagar University, Savar, Dhaka-1342, Bangladesh; 3 Laboratory of Research on Local Animal Products, Ibn Khaldoun University, Tiaret 14000, Algeria; 4 Human Genome Centre, School of Medical Sciences, Universiti Sains Malaysia, Kubang Kerian 16150, Kelantan, Malaysia

**Keywords:** Algerian honey, physicochemical properties, DPPH, FRAP, proline, ascorbic acid

## Abstract

The aim of the present study was to characterize the physical, biochemical and antioxidant properties of Algerian honey samples (n = 4). Physical parameters, such as pH, moisture content, electrical conductivity (EC), total dissolved solids (TDS), color intensity, total sugar and sucrose content were measured. Several biochemical and antioxidant tests were performed to determine the antioxidant properties of the honey samples. The mean pH was 3.84 ± 0.01, and moisture the content was 13.21 ± 0.16%. The mean EC was 0.636 ± 0.001, and the mean TDS was 316.92 ± 0.92. The mean color was 120.58 ± 0.64 mm Pfund, and the mean 5-hydroxymethylfurfural (HMF) content was 21.49 mg/kg. The mean total sugar and reducing sugar contents were 67.03 ± 0.68 g/mL and 64.72 ± 0.52 g/g, respectively. The mean sucrose content was 2.29 ± 0.65%. High mean values of phenolic (459.83 ± 1.92 mg gallic acid/kg), flavonoid (54.23 ± 0.62 mg catechin/kg), ascorbic acid (159.70 ± 0.78 mg/kg), AEAC (278.15 ± 4.34 mg/kg), protein (3381.83 ± 6.19 mg/kg) and proline (2131.47 ± 0.90) contents, as well as DPPH (39.57% ± 4.18) and FRAP activities [337.77 ± 1.01 µM Fe (II)/100 g], were also detected, indicating that Algerian honey has a high antioxidant potential. Strong positive correlations were found between flavonoid, proline and ascorbic acid contents and color intensity with DPPH and FRAP values. Thus, the present study revealed that Algerian honey is a good source of antioxidants.

## 1. Introduction

Honey is a sweet and flavorful product that has been consumed over the years for its high nutritional values and beneficial effects on human health. According to a previous report [1], the chemical composition of honey is complex, containing approximately 181 substances including sugars, proteins, moisture, vitamins, minerals, 5-hydroxymethylfurfural (HMF), enzymes, flavonoids, phenolic acids and volatile compounds. The main constituents of honey are moisture, glucose, fructose, sucrose, minerals and proteins [2,3].

The quality of honey is mainly determined by its sensorial, chemical, physical and microbiological characteristics. The criteria for ensuring quality honey have been specified by the EC Directive 2001/110 [4]. The major criteria are moisture content, electrical conductivity, ash content, reducing and non-reducing sugars, free acidity, diastase activity and HMF content [2,5].

Honey contains a number of compounds and the antioxidant properties of honey are well known. The antioxidant properties of honey are derived from both enzymatic (e.g., catalase, glucose oxidase and peroxidase) and nonenzymatic substances (e.g., ascorbic acid, α-tocopherol, carotenoids, amino acids, proteins, Maillard reaction products, flavonoids and phenolic acids) [5,6,7]. The amount and type of these antioxidants are largely dependent on the floral source or honey variety, and a correlation between antioxidant activity with total phenolic content has been established [5,7].

Although honey is widely consumed in Algeria, there is still a lack of information on the physicochemical and antioxidant properties of Algerian honeys. To date, some physicochemical properties of Algerian honeys, such as pH, moisture content, electrical conductivity, total sugar content and proline have been reported [8,9], but other important physicochemical properties, such as HMF, sucrose and protein contents, as well as antioxidant properties (phenolics, flavonoids, ascorbic acid, DPPH, FRAP, AEAC), have never been reported.

## 2. Results and Discussion

### 2.1. Physical Analyses

#### 2.1.1. pH of Honey

Honey is naturally acidic irrespective of its geographical origin, which may be due to the presence of organic acids that contribute to its flavor and its stability against microbial spoilage. The pH of honey samples is important during the extraction process because it affects the texture of honey as well as its stability and shelf life [10].

All of the tested Algerian honey samples were acidic in nature, with pH values that varied between 3.70 and 4.00 (Table 1). These values were similar to those previously reported for other honey samples from India, Brazil, Spain and Turkey, which were reported to have pHs between 3.49 and 4.70 [11,12,13]. The pH values of Algerian honey samples have been previously reported to be 3.49–4.43 [9] and 3.29–4.37 [8]. A highly acidic honey sample indicates the possible fermentation of sugars into organic acids. None of the investigated samples exceeded the allowed limit, which may be considered as an index of freshness of all honey samples.

**Table 1:** Physical parameters (pH, moisture content, EC, TDS, color characteristics and HMF concentrations) of Algerian honey.

Sample	pH	Moisture content (%) mean ± SD	Electrical Conductivity (EC) mean ± SD mS/cm	Total dissolved solids (TDS) mean ± SD ppm	HMF (mg/kg) mean ± SD	ABS_450_ (mAU; 50 w/v) mean ± SD
AH-1	3.70 ± 0.0 ^d^	13.73 ± 0.12 ^b^	0.417 ± 0.0006 ^d^	208.0 ± 1.00 ^d^	22.60 ± 0.02 ^c^	724.00 ± 2.00 ^d^
AH-2	3.87 ± 0.06 ^b^	11.59 ± 0.31 ^c^	0.806 ± 0.0012 ^a^	399.3 ± 1.53 ^a^	24.21 ± 0.16 ^a^	873.67 ± 2.52 ^c^
AH-3	4.00 ± 0.00 ^a^	14.13 ± 0.12 ^a^	0.764 ± 0.0023 ^b^	381.7 ± 0.58 ^b^	23.93 ± 0.29 ^b^	1103.00 ± 3.61 ^b^
AH-4	3.80 ± 0.00 ^c^	13.39 ± 0.12 ^b^	0.558 ± 0.0006 ^c^	278.7 ± 0.58 ^c^	15.23 ±0.14 ^a^	1188.00 ± 1.73 ^a^
Mean	3.84 ± 0.01	13.21 ± 0.16	0.636 ± 0.001	316.92 ± 0.92	21.49 ± 0.15	972.16 ± 2.46

Means are compared by using One way ANOVA-*Post Hoc* Multiple Comparisons; in each column, values with different letters (superscripts) indicate significant differences (*p <* 0.05).

#### 2.1.2. Moisture Content

The moisture content in the investigated honey samples was between 11.59 and 14.13%, which are within the limit (≤20%) recommended by the international quality regulations [4,14] (Table 1). Water content is very important for the shelf life of honey during storage [15] and can lead to undesirable honey fermentation due to osmotolerant yeasts, which form ethyl alcohol and carbon dioxide [16]. Generally, all of the investigated Algerian honey samples were of good quality, as indicated by the low moisture content.

#### 2.1.3. Total Sugar Content

The total sugar content of the honey tested was similar with the findings of other previously studied Algerian honeys [9]. None of the samples exceeded the highest limit set for total sugar content by the European community directive [4] (Table 2).

**Table 2:** Reducing and non-reducing sugar content of Algerian honey.

Sample	Total sugar content mean ± SD% (g/mL)	Reducing sugar mean ± SD (%) g/g	Sucrose mean ± SD (%)
**AH-1**	62.80 ± 1.06 ^a^	60.19 ± 0.70 ^a^	2.54 ± 0.71 ^a^
**AH-2**	65.73 ± 0.46 ^b^	63.94 ± 0.67 ^b^	1.80 ± 0.84 ^a^
**AH-3**	69.60 ± 0.40 ^a^	67.08 ± 0.55 ^a^	2.52 ± 0.37 ^a^
**AH-4**	70.00 ± 0.80 ^a^	67.70 ± 0.18 ^a^	2.30 ± 0.69 ^a^
**Mean**	67.03 ± 0.68	64.72 ± 0.52	2.29 ± 0.65

Means are compared by using One way ANOVA-Post Hoc Multiple Comparisons. In each column, values with different letters (superscripts) indicate significant differences (*p<* 0.05).

#### 2.1.4. Electrical Conductivity and Total Dissolved Solids

EC is one of the most important factors for determining the physical characteristics of honey [17]. It is also an important physicochemical measurement for the authentication of uniﬂoral honeys [18]. With the exception of a single sample (0.806 mS/cm), the EC values of samples were within the allowed parameters (lower than 0.8 mS/cm) (Table 1). The values of EC change when the amount of plant pollen decreases. According to Persano *et al.* [19], the nectars from some plants are “stronger” than others, and even low contamination of honey with “stronger” nectar can modify its sensory and physicochemical properties. The EC values of some Algerian honeys were reported to be 0.21–1.61 mS/cm in a previous study by Ouchemoukh *et al.* [9]. However, our results are similar to the findings previously reported by Saxena *et al.* [20] and Alvarez-Suarez *et al.* [21].

TDS is a measure of the combined content of all inorganic and organic substances in honey in the molecular, ionized or micro-granular (colloidal solution) suspended forms. Our results demonstrate that there is a good correlation between EC and TDS, indicating that both parameters can be used to determine honey purity.

#### 2.1.5. Color Characteristics

**Figure 1:**
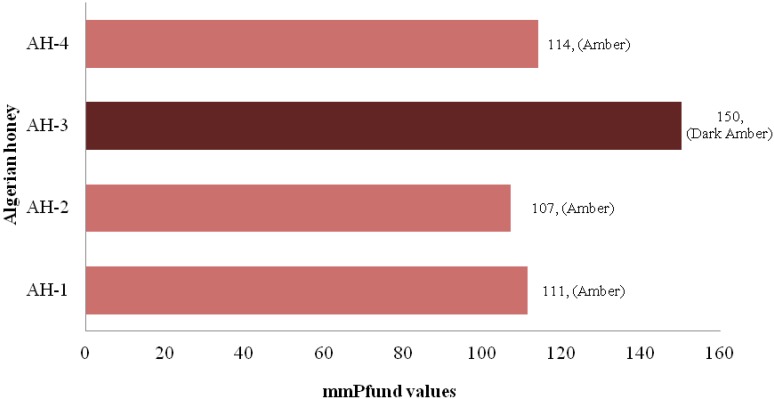
Color characteristics of Algerian honey.

The primary characteristic for honey classification is color, which is classified according to USDA-approved color standards [22]. Honey color varies naturally in a wide range of tones, ranging from light yellow to amber, dark amber and black, in extreme cases, and sometimes even green or red hues may occur [23]. The color of untreated honey depends on its botanical origins. For this reason, color is very important for the classification of monofloral honeys for commercial activities. Honey darkens with age, and other changes in color may result from the beekeeper’s interventions and different ways of conservation, such as the use of old honeycombs, contact with metals, and exposure to high temperatures or light. One of the Algerian honey samples (AH-3) was dark amber in color and has the highest Pfund value, whereas the other three honey samples were amber in color (Figure 1).

#### 2.1.6. Color intensity (ABS_450_)

ABS_450_ is related to the presence of pigments, such as carotenoids and flavonoids, which are known to have antioxidant properties [24]. ABS_450_ values of the investigated samples ranged from 724 to 1188 mAU (Table 1). This is the first ABS_450_ report for Algerian honey. The reported ABS_450_ values for some Italian, Slovenian and Indian honeys were reported to be 25–3413 mAU, 70–495 mAU and 524–1678 mAU, respectively [13,25,26].

#### 2.1.7. Determination of HMF Concentrations by HPLC Method

HMF formation results from the acid-catalyzed dehydration of hexose sugars with fructose being particularly susceptible. In addition, HMF is only present in trace amounts in fresh honey, and its concentration has been reported to increase with storage and the prolonged heating of the honey. HMF is thus an essential parameter used to indicate honey purity. With the exception of a single sample (AH-4) that contained 15.23 mg/kg of HMF, the HMF concentrations of the remaining honey samples were similar, ranging from 22.60 to 24.21 mg/kg (Table 1). Notably, all HMF concentrations were within the recommended range set by the Codex Alimentarius [27] at 80 mg/kg. The values are also within the allowed maximum limit of 40 mg/kg, as recommended by the Turkish Alimentarus Codex [28] for honey samples from tropical countries.

We compared our results with honey samples from different countries. Persano *et al.* [29] reported low HMF concentrations of two unprocessed Australian honey samples, Grey box and Banksia (1.35 and 1.12 mg/kg, respectively). The HMF concentrations of some Australian honeys, such as rainforest, Homebrand and Mallee honey, were reported to be 2.2, 17.7 and 34.0 mg/kg, respectively [29,30]. High HMF formation may occur due to overheating, exposure to high temperatures [29] or the type of sugar present in the honey, as well as the fructose/glucose ratio [31]. Overall, the low HMF concentrations of the tested Algerian honey confirm that these samples are of good quality.

### 2.2. Antioxidant Analyses

#### 2.2.1. Polyphenol Content

The antioxidant activity of natural honeys depends largely on their chemical composition, such as phenolics, flavonoids, enzymes, organic acids, amino acids, Maillard reaction products, ascorbic acid, carotenoids, as well as their origins [32,33]. Thus, phenolics or polyphenols are one of the most important classes of compounds found in honey. The total concentration of phenols in honey is highly dependent on its plant source. High concentrations of polyphenols were found in all of our honey samples (Table 3). The highest concentration of polyphenols was determined to be 498.16 mg/kg for sample AH-4, indicating its high antioxidant potential.

Because the content of phenolic compounds is usually lower in light-colored honey compared to that of dark honeys, the high levels of polyphenols in all honeys may contribute to its darker color [34]. All of our honey samples were amber to dark amber in color. The total polyphenol content of the tested Algerian honey is higher than those of two reported Malaysian honey samples, as well as that of Gelam and Coconut honeys [35], which are lighter in color. In addition, the phenolic content of the four honey samples analyzed were higher than those of natural honeys [1,26,32,36,37]. The high level of polyphenols in the studied Algerian honeys further indicates their high antioxidant properties.

#### 2.2.2. Flavonoid Content

Flavonoids are low molecular weight phenolic compounds that are vital components for the aroma and antioxidant properties of honey. Flavonoid content is expressed as mg of catechin per kg of honey. Similar to polyphenol content, the AH-4 sample contained the highest amount (71.78 mg/kg) of flavonoids (Table 3). This result is similar to a previous study in which honey samples with high polyphenol concentrations also contained high flavonoid levels [38]. The flavonoid content of these honeys is higher than that of Turkish [39] and Malaysian honeys, which range from 4.80 to 22.80 mg/kg and 11.52–25.31 mg/kg, respectively [38], indicating that Algerian honey has a higher antioxidant potential.

**Table 3:** Biochemical and antioxidant properties of Algerian honey.

Sample	Total polyphenols mean ± SD (mg_gallic acid_/kg)	Flavonoids mean ± SD (mg_catechin_/kg)	FRAP values mean ± SD (µM Fe (II)/100 g)	Proline mean ± SD (mg/kg)	Protein mean ± SD (mg/kg)
**AH-1**	411.10 ± 1.55 ^d^	27.07 ± 0.35 ^d^	287.45 ± 0.92 ^d^	1692.18 ± 1.00 ^d^	3007.33 ± 3.54 ^d^
**AH-2**	483.01 ± 2.15 ^b^	52.24 ± 0.03 ^c^	306.60 ± 1.16 ^c^	1946.01 ± 0.84	3031.67 ± 10.61 ^c^
**AH-3**	447.06 ± 2.67 ^c^	65.85 ± 1.31 ^b^	353.50 ± 0.65 ^b^	2175.31 ± 0.78 ^b^	3393.33 ± 7.07 ^b^
**AH-4**	498.16 ± 1.32 ^a^	71.78 ± 0.84 ^a^	403.54 ± 1.31 ^a^	2712.39 ± 0.98 ^a^	4095.00 ± 3.54 ^a^
**Mean**	459.83 ± 1.92	54.23 ± 0.62	337.77 ± 1.01	2131.47 ± 0.90	3381.83 ± 6.19

Means are compared by using One way ANOVA-*Post Hoc* Multiple Comparisons. In each column, values with different letters (superscripts) indicate significant differences (*p <* 0.05).

#### 2.2.3. DPPH free Radical-Scavenging Activity

The radical scavenging activities of honey samples were measured using the DPPH radical scavenging assay. DPPH is a stable nitrogen-centered radical that has been extensively used to test the free radical scavenging ability of various samples. In evaluating the radical-scavenging potential of honeys, the DPPH assay is frequently used because the antioxidant potential of honey has been shown to be directly associated with its phenolic and flavonoid contents [25]. High DPPH scavenging activity confers the superior antioxidant activity of the sample.

**Figure 2:**
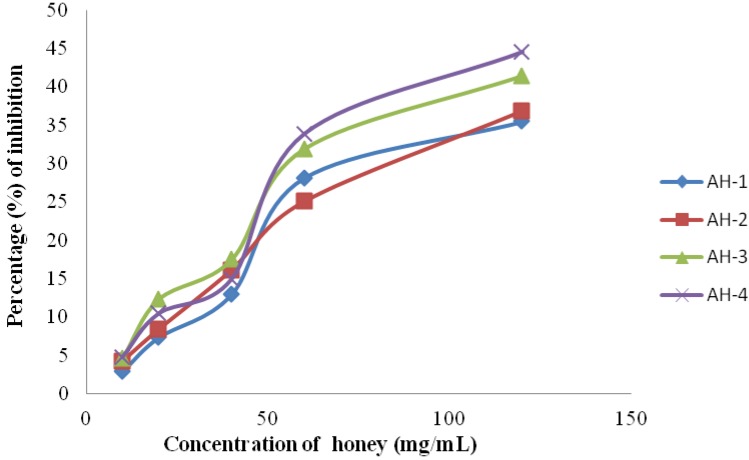
Percentage of inhibition of DPPH radical scavenging activity at different concentrations of Algerian honey.

The DPPH radical scavenging activities of all honey samples were measured at the following concentrations: 10, 20, 40, 60 and 120 mg/mL. The highest percentage of inhibition was observed at 120 mg/mL for all honey samples. The highest percentage of inhibition was exhibited by the AH-4 sample (44.57%), thus indicating its high antioxidant potential (Figure 2). The percentage of inhibition exhibited by Algerian honey is similar to that of some Malaysian [40] and Indian honey samples [13].

#### 2.2.4. Determination of Total Antioxidant Content by FRAP Assay

To determine the antioxidant capacity of the honeys studied, a FRAP assay, which is a simple direct test widely used for the determination of antioxidant activity in many different samples, including honey [25,35,37,41,42,43], was used. Again, the AH-4 sample exhibited the highest FRAP values (403.54 ± 1.31 µM Fe (II)/100 g), confirming its high antioxidant properties (Table 3). These are the first FRAP values published for Algerian honey samples. High FRAP values indicate a greater reduction of ferric ions to ferrous ions. Samples with a higher reducing power increased in absorbance at 700 nm.

#### 2.2.5. Proline Content

Proline is an important amino acid that originates mostly from the salivary secretions of *Apis mellifera* during the conversion of nectar into honey [44]. Proline content is an indication of honey ripeness and, in some cases, sugar adulteration. Proline was detected in high concentrations (1692–2712 mg/kg) in all of Algerian honey samples tested. Some authors have reported that high concentrations of proline are also typical for honeydew honeys [9,23,32].

#### 2.2.6. Ascorbic Acid and AEAC Assay

**Figure 3:**
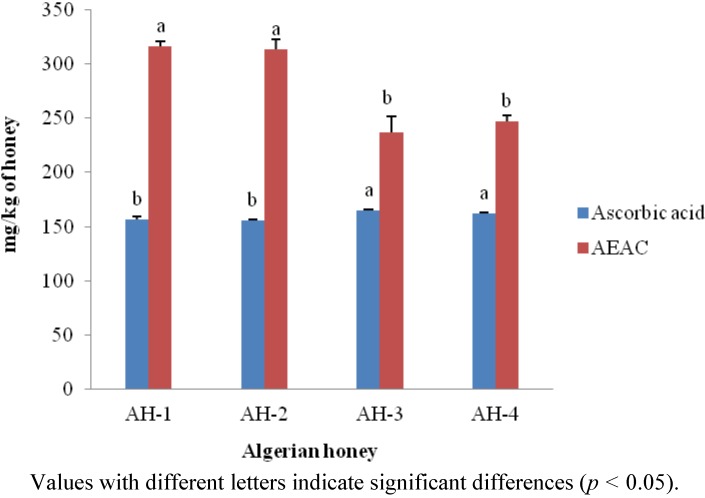
Ascorbic acid and AEAC contents of Algerian honey.

The AEAC content of Algerian honey samples was measured in mg of AEAC/100 g of honey using an ascorbic acid standard curve (r^2^ = 0.9447). Algerian honey samples exhibited AEAC values ranging from 236.80 to 315.90 mg of AEAC/kg (Figure 3). These values are similar to those of honeys from Burkina Fasan [32], whereas Indian honey samples exhibited lower values (between 151 and 295 mg of AEAC/kg) [13].

To our knowledge, this is the first report on the ascorbic acid content and AEAC values of Algerian honeys. A negative correlation was observed between ascorbic acid and AEAC values in honey samples that contained higher ascorbic acid and had lower AEAC values. Thus, the high ascorbic acid content contributes to the reduction in DPPH free radical activity.

### 2.3. Biochemical Analyses

#### 2.3.1. Reducing Sugar Content

The total reducing sugar content in honey samples ranged from 62.80 to 70.00% (Table 2). Our data indicate that reducing sugars are the major soluble sugars present in Algerian honey samples. With respect to reducing sugars (fructose and glucose), the EC Directive 2001/110 imposes that the amount of reducing sugars should be ≥60 g/100 g, with the exception of honeydew honey, which has a lower limit (≥45 g/100 g). Our results meet this standard and are similar to other published levels for reducing sugars [13,45].

The amount of non-reducing sugars, such as sucrose content (%), was measured by subtracting the reducing sugar content from total sugar content, which is expressed by the following equation:


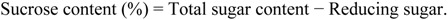


The sucrose content in Algerian honey samples ranged from 1.80 to 2.54%, which is below 5% or the maximum prescribed limit set by the Codex standard [14].

#### 2.3.2. Protein Content

The concentrations of proteins and amino acids in honeys vary depending on their botanical or geographical origin and storage time. Protein content in honey samples are reported to consist of mainly enzymes [3]. Different enzymes are also added by bees during the process of honey ripening, indicating that the colorimetric determination of the protein content of honey samples using the method of Lowry is suitable. High protein content (4,097.00 ± 3.54 mg/kg) was found in AH-4 when compared to the mean protein content of other analyzed honeys. Protein content in honey generally ranges from 2,000 to 5,000 mg/kg [46].

### 2.4. Correlation amongst Biochemical Parameters and Antioxidant Properties

The correlation matrix (Table 4) indicates a significant correlation between biochemical and antioxidant parameters. A strong correlation was found between the color intensity of honey samples and the antioxidant parameters, flavonoid and proline contents, as well as the DPPH and FRAP values, at 0.968, 0.934, 0.964, 0.963, respectively. Thus, color pigments may have a role in the observed antioxidant activities of honey samples. The color intensity also increases with increases in the phenolic and flavonoid contents of honey. A strong correlation between ABS_450_, DPPH and FRAP values suggest the involvement of pigments that confer antioxidant properties to honey. A study conducted by Terrab *et al.* [26] also found a strong correlation between ABS_450_ and FRAP values (r = 0.85) in Slovenian honeys. In Indian honeys, the correlation between ABS_450_ and FRAP values was 0.83 [13]. Thus, the higher correlation in our study indicates that Algerian honeys have a stronger antioxidant capacity compared to Indian honeys.

**Table 4:** Correlation matrix showing the interrelation among phenolics, flavonoids, DPPH scavenging, FRAP, ascorbic acid, proline, ABS_450_ and protein.

	Phenolics	Flavonoids	DPPH	FRAP	Ascorbic acid	Proline	ABS_450_	Protein
**Phenolics**	1.000	0.776 **	0.615 *	0.668 *	0.165	0.764 **	0.662 *	0.629 *
**Flavonoids**	0.776 **	1.000	0.888 **	0.893 **	0.730 **	0.887 **	0.968 **	0.778 **
**DPPH**	0.615 *	0.888 **	1.000	0.982 **	0.785 **	0.956 **	0.964 **	0.940 **
**FRAP**	0.668 *	0.893 **	0.982 **	1.000	0.749 **	0.987 **	0.963 **	0.973 **
**Ascorbic acid**	0.165	0.730 **	0.785 *	0.749 **	1.000	0.644 *	0.828 **	0.646 *
**Proline**	0.764 **	0.887 **	0.956 **	0.987 **	0.644 *	1.000	0.934 **	0.974 **
**ABS_450_**	0.662 *	0.968 **	0.964 **	0.963 **	0.828 **	0.934 **	1.000	0.876 **
**Protein**	0.629 *	0.778**	0.940 **	0.973**	0.646 *	0.974 **	0.876 **	1.000

** Correlation is significant at the 0.01 level (2-tailed); * Correlation is significant at the 0.05 level (2-tailed).

A positive significant linear correlation was observed between the following antioxidant parameters: flavonoid content with DPPH RSA (r = 0.888) and FRAP values (r = 0.893). Similar correlations between DPPH RSA and flavonoid content (r = 0.888) were observed in some Malaysian honey samples [38]. However, the correlations between phenolics with DPPH radical scavenging activity (r = 0.615) and FRAP values (r = 0.668) were lower than with flavonoids. The correlation between DPPH RSA and total phenolic content suggest that phenolics were the strongest contributing factor to the RSA of these honeys compared to FRAP.

Proline is an important amino acid that confers antioxidant properties to honey and strongly correlates with DPPH, FRAP and ABS_450_. The most significant correlation was observed between proline content and FRAP values (r = 0.987), which is higher than those values reported by Diez *et al*. [32], indicating that proline content also contributes to the antioxidant potential of Algerian honey. The correlation between DPPH scavenging activity and proline content was 0.956. However, in another report, the correlation between proline content and DPPH RSA was lower (0.75) [32], even though the correlation in Indian honey samples (r = 0.94) was similar to our results [13]. Thus, as with Indian honey samples, the total proline content in the honey samples in this study may be a critical factor responsible for the antioxidant activity of Algerian honey. However, in future, it will be good to confirm these findings with larger number of samples.

To summarize, proline content may be a significant determinant of the antioxidant capacity of Algerian honey samples as well as their reducing ability and radical scavenging potential. Protein content was also strongly correlated with proline content (r = 0.974), FRAP (r = 0.973) and DPPH RSA (r = 0.940) values. Ascorbic acid is an important vitamin that is well known for its antioxidant properties. It was observed to significantly correlate with flavonoids (r = 0.730), DPPH RSA (r = 0.785) and FRAP (r = 0.749). The best correlation between ascorbic acid and ABS_450_ was observed at r = 0.828, indicating that similar to ascorbic acid content, color pigments are good indicators of antioxidant potential. These correlations demonstrate that the overall antioxidant property in the investigated Algerian honeys can be attributed to various factors, such as proline, phenolic, flavonoid and ascorbic acid contents, as well as color pigments. Furthermore, proline content appears to be highly important for antioxidant activity as shown by the correlation values.

## 3. Experimental

### 3.1. Honey Samples

Four local Algerian honey samples (AH1, AH2, AH3 and AH4) were purchased from supermarket shelves in the capital of Algeria between May 2010 and July 2010. All of the honey samples were stored at room temperature (22–24 °C) in airtight plastic containers until analysis.

### 3.2. Chemicals and Reagents

Ascorbic acid, bovine serum albumin (BSA), catechin, 2,2-diphenyl-1-picrylhydrazyl (DPPH), 2,4,6-tris(1-pyridyl)-1,3,5-triazine (TPTZ), HMF, Folin–Ciocalteu’s reagent, gallic acid and proline were purchased from Sigma-Aldrich (St. Louis, MO, USA). Sodium carbonate (Na_2_CO_3_), aluminum chloride (AlCl_3_), sodium nitrite (NaNO_2_) and sodium hydroxide (NaOH) were purchased from Merck (Darmstadt, Germany). All chemicals used were of analytical grade.

### 3.3. Physical Analysis

#### 3.3.1. pH

A pH meter (HI 98127, Hanna instruments, Mauritius) was used to measure the pH of a 10% (w/v) solution of honey prepared in milli-Q water (Millipore Corporation, Billerica, MA, USA).

#### 3.3.2. Moisture Content

The moisture content was determined using a refractometric method. In general, the refractive index increases with increases in the solid content of a sample. The refractive indices of honey samples were measured at ambient temperature using an Atago handheld refractometer (KRUSS, HRH30, Hamburg, Germany), and measurements were further corrected for the standard temperature of 20 °C by the addition of the 0.00023/°C correction factor. The moisture content was measured in triplicate, and the percentage of moisture content, which corresponds to the corrected refractive index, was calculated using Wedmore’s table [47].

#### 3.3.3. Total Sugar Content

Honey was suspended in milli-Q water to make a 25% (w/v) solution. The total sugar content of each honey sample was determined using the refractometric method (Atago handheld refractometer, ATAGO, N-1α, Tokyo, Japan). The percentage of sucrose content was measured in g/mL of honey.

#### 3.3.4. Electrical Conductivity (EC) and Total Dissolved Solids (TDS)

EC and TDS were measured using a conductivity meter HI 98311 (Hanna Instruments, Mauritius) for a 20% (w/v) solution of honey suspended in milli-Q water [16]. The EC of milli-Q water was determined to be less than 10 µS/cm. The EC and TDS of each sample was analyzed in triplicate, and the means were expressed in mS/cm and ppm, respectively.

#### 3.3.5. Honey Color Analysis

The color intensity of honey samples was measured according to the Pfund classifier. Briefly, homogeneous honey samples devoid of air bubbles were transferred into a cuvette with a 10-mm light path to approximately half cuvette full. The cuvette was inserted into a color photometer (HI 96785, Hanna Instruments, Cluj County, Romania). Color grades were expressed in millimeter (mm) Pfund grades when compared to an analytical grade glycerol standard. Measurements were performed in triplicate for each sample using approved color standards of the United States Department of Agriculture (USDA) [22].

#### 3.3.6. Color Intensity (ABS_450_)

The mean absorbance of honey samples was determined using the method of [25]. Briefly, honey samples were diluted to 50% (w/v) with warm (45–50 °C) milli-Q water and the resulting solution was filtered using a 0.45 µm filter to remove large particles. The absorbance was measured at 450 and 720 nm using a spectrophotometer, and the difference in absorbance was expressed as mAU.

#### 3.3.7. Determination of HMF by High-Performance Liquid Chromatography (HPLC) Method

HMF concentrations were determined using an HPLC method based on the method published by the International Honey Commission (IHC) [48]. Briefly, honey samples (10 g each) were diluted to 50 mL with distilled water, filtered using a 0.45 μm nylon membrane filter and injected (20 μL) into an HPLC system (Waters 2695, Milford, MA, USA) equipped with a Photodiode Array Detector (PDA) (Waters 2996). The HPLC column used was a Merck Purospher Star RP-18e (125 × 4 mm, 5 μm) fitted with a guard cartridge packed with similar stationary phase (Merck, Germany). The HPLC method included an isocratic mobile phase of 90% water and 10% methanol with a flow rate of 1.0 mL/min. All solvents used were of HPLC grade. The detection wavelength was 200–450 nm with specific monitoring at 285 nm. HMF concentrations in samples were calculated by comparing the corresponding peak areas of the sample and HMF standard solutions (Sigma-Aldrich) after correcting for the dilution of honey samples. A linear relationship (r^2^ = 0.9997) was determined between the concentration and area of HMF peaks (results are expressed in mg/kg).

### 3.4. Analysis of Antioxidant Properties

#### 3.4.1. Determination of Total Phenolic Content

The concentration of phenolics in honey samples was estimated using a modified spectrophotometric Folin-Ciocalteu method [49]. Briefly, 1 mL of honey extract was mixed with 1 mL of Folin-Ciocalteu phenol reagent. After 3 min, 1 mL of 10% Na_2_CO_3_ solution was added to the mixture and adjusted to 10 mL with distilled water. The reaction was kept in the dark for 90 min, after which the absorbance was read at 725 nm using a T 60 UV/VIS spectrophotometer (PG Instruments Ltd, London, UK). Gallic acid was used to calculate a standard curve (20, 40, 60, 80 and 100 μg/mL; r^2^ = 0.9970). The concentration of phenolic compounds was measured in triplicate. The results were reported as the mean ± standard deviation and expressed as mg of gallic acid equivalents (GAEs) per kg honey.

#### 3.4.2. Determination of Total Flavonoid Content

The total flavonoid content in each honey sample was measured using the colorimetric assay developed by Bergner *et al.* [50]. Honey extract (1 mL) was mixed with distilled water (4 mL). At the baseline, NaNO_2_ (5%, w/v, 0.3 mL) was added. After five min, AlCl_3_ (10% w/v, 0.3 mL) was added followed by the addition of NaOH (1 M, 2 mL) 6 min later. The volume was then increased to 10 mL by the addition of distilled water (2.4 mL). The mixture was vigorously shaken to ensure adequate mixing, and the absorbance was read at 510 nm. A calibration curve was created using a standard solution of catechin (20, 40, 60, 80 and 100 μg/mL; r^2^ = 0.9880). The results were expressed as mg catechin equivalents (CEQ) per kg of honey.

#### 3.4.3. DPPH Free Radical-Scavenging Activity

The antioxidant properties of each honey sample were also studied by evaluating the free radical-scavenging activity of the DPPH radical, which was based on the method proposed by Ferreira *et al.* [6]. Briefly, honey extract (0.5 mL) was mixed with methanolic solution containing DPPH radicals (0.024 mg/mL, 2.7 mL). The mixture was vigorously shaken and left to stand for 15 min in the dark (until their absorbance remained unchanged). The reduction of the DPPH radical was determined by measuring the absorbance of the mixture at 517 nm [51].

Butylated hydroxytoluene (BHT) was used as a reference. The radical-scavenging activity (RSA) was calculated as the percentage of DPPH discoloration using the following equation: % RSA = ([A_DPPH_ – A_S_]/A_DPPH_) × 100, where A_S_ is the absorbance of the solution when the sample extract has been added at a particular level and A_DPPH_ is the absorbance of the DPPH solution.

#### 3.4.4. Ferric Reducing/Antioxidant Power Assay (FRAP Assay)

The FRAP assay was performed according to a modified method described by Benzie and Strain [41]. Briefly, properly diluted honey (0.1 g/mL, 200 μL) was mixed with FRAP reagent (1.5 mL). Then, the reaction mixture was incubated at 37 °C for 4 min and its absorbance was read at 593 nm against a blank that was prepared with distilled water. Fresh FRAP reagent was prepared by mixing 10 volumes of 300 mM/L acetate buffer (pH 3.6) with 1 volume of 10 mmol TPTZ solution in 40 mM/L HCl containing 1 volume of 20 mM ferric chloride (FeCl_3_.6H_2_O). The resulting mixture was then pre-warmed at 37 °C. A calibration curve was prepared using an aqueous solution of ferrous sulfate (FeSO_4_.7H_2_O) at 100, 200, 400, 600 and 1,000 μM/L. FRAP values were expressed as micromoles of ferrous equivalent (μM Fe [II]) per kg of honey.

#### 3.4.5. Determination of Ascorbic Acid Content

The ascorbic acid content was measured using the method described by Ferreira *et al.* [6]. Briefly, sample (100 mg) was extracted with 1% metaphosphoric acid (10 mL) at room temperature for 45 min and filtered through Whatman No. 4 filter paper. The filtrate (1 mL) was mixed with 0.005% 2,6-dichlorophenolindophenol (DCPIP, 9 mL), and the absorbance of the mixture was measured within 30 min at 515 nm against a blank. The ascorbic acid content was calculated based on a calibration curve of authentic L-ascorbic acid (50, 100, 200 and 400 µg/mL; Y = 3.2453X − 0.0703; r*^2^* = 0.9440). The results were expressed as mg of ascorbic acid/kg of honey.

#### 3.4.6. Antioxidant Content

Antioxidant content was determined by measuring AEAC values using the method of Meda *et al.* [32]. Briefly, honey samples were dissolved in methanol to a final concentration of 0.03 g/mL. A 0.75-mL aliquot of the methanolic honey solution was then mixed with a 0.02 mg/mL DPPH solution prepared in methanol (1.50 mL). The mixture was incubated at room temperature for 15 min, and the absorbance was measured at 517 nm using a spectrophotometer. The blank was composed of a methanolic honey solution (0.75 mL of 0.03 g/mL honey) mixed with methanol (1.5 mL). Ascorbic acid standard solutions (1, 2, 4, 6 and 8 µg/mL) prepared in milli-Q water were used to form a calibration curve. Measurements were performed in triplicate, and the mean value was expressed as mg of ascorbic acid equivalent antioxidant content per 100 g of honey.

#### 3.4.7. Proline Content

Proline content in honey samples was measured using a method established by the IHC [52]. Briefly, BSA solutions were prepared by diluting a stock solution of BSA (1 mg/mL) to 5 mL. BSA concentrations ranged from 0.05 to 1.00 mg/mL. From the dilutions, 0.2 mL of protein solution were transferred to different test tubes and 2 mL of alkaline copper sulfate reagent (analytical reagent) were added before proper mixing. The resulting solution was incubated at room temperature for 10 min. Then, 0.2 mL of Folin-Ciocalteau solution was added to each tube and incubated for 30 min. The absorbance was measured at 660 nm.

### 3.5. Biochemical Analyses

#### 3.5.1. Protein Content

The protein content of honey was measured according to Lowry’s method [52]. Briefly, BSA solutions were prepared by diluting a stock BSA solution (1 mg/mL) to 5 mL. BSA concentrations ranged from 0.05 to 1.00 mg/mL. Based on these different dilutions, 0.2 mL of protein solution was placed in different test tubes and 2 mL of alkaline copper sulfate reagent (analytical reagent) was added. After the resulting solution was mixed properly, it was incubated at room temperature for 10 min. Then, 0.2 mL of reagent Folin-Ciocalteau solution was added to each tube and incubated for 30 min. The colorimeter was calibrated with a blank, and the absorbance was measured at 660 nm.

#### 3.5.2. Reducing Sugar Assay

The total reducing sugar content was measured using 3,5-dinitrosalicylic acid (DNSA). In principle, the reducing sugar reduces DNSA to 3-amino-5-nitrosalicylic acid, resulting in a solution with reddish-orange coloration, which is measured spectrophotometrically at 540 nm [13]. The honey solution (0.1 g/mL) was diluted 100-fold with milli-Q water. A 1-mL aliquot of this diluted solution was mixed with equal amounts of DNSA solution and incubated in a boiling water bath for 10 min. The mixture was allowed to cool to ambient temperature for 10 min, mixed with 7.5 mL of milli-Q water and the absorbance was measured at 540 nm using a spectrophotometer. A glucose solution of known concentration (100, 200, 400 and 600 µg/mL) was used as a standard.

### 3.6. Statistical Analyses

Assays were performed in triplicate, and the results were expressed as mean values with standard deviations (SD). The significant differences represented by letters were obtained by a one-way analysis of variance (ANOVA) followed by Tukey’s honestly significant difference (HSD) *post hoc* test (*p* < 0.05). Correlations were established using Pearson’s correlation coefficient (r) in bivariate linear correlations (*p* < 0.01). These correlations were calculated using Microsoft office Excel 2007 and SPSS version 18.0 (SPSS Inc., Chicago, IL, USA).

## 4. Conclusions

This is the first study to investigate the physicochemical and antioxidant properties of Algerian honeys more elaborately. This study showed that Algerian honey samples have high antioxidant potential, as indicated by their high phenolic, flavonoid, ascorbic acid and proline contents. Furthermore, several strong positive correlations were observed amongst the different antioxidant markers and antioxidant test values. These correlations demonstrate that the overall antioxidant property in the Algerian honeys studied can be attributed to various contributing factors, such as proline, phenolic, ascorbic acid and flavonoid contents, as well as color pigments, and the relevance of proline appears to be prominent, as indicated by its correlation values. Our results indicate that Algerian honey is a good source of antioxidants.
